# Ecological system theory and community participation to promote healthy food environments for obesity and non-communicable diseases prevention among school-age children

**DOI:** 10.1017/S136898002300040X

**Published:** 2023-07

**Authors:** Pennapa Ritwong Suwannawong, Naruemon Auemaneekul, Arpaporn Powwattana, Rewadee Chongsuwat

**Affiliations:** 1 Department of Public Health Nursing, Faculty of Public Health, Mahidol University, 420/1 Ratchawithi Road, Thung Phaya Thai, Ratchathewi District, Bangkok 10400, Thailand; 2 Department of Nutrition, Faculty of Public Health, Mahidol University, Bangkok, Thailand

**Keywords:** Obesity prevention, Participation, Ecological system theory, School-age children, Healthy foods

## Abstract

**Objectives:**

To implement and evaluate the effectiveness of the community participatory program between school and family based on ecological system theory and participatory action research. The intervention covers three levels at the individual, family and school levels and involves educating students and parents by using technology, reducing sedentary behaviours, increasing exercise and changing to healthy food environments at school and at home.

**Design:**

A quasi-experimental design was used in this study.

**Setting:**

Public primary school in Thailand.

**Subjects:**

The participants in the study included 138 school-age children in grades 2–6 with their parents/guardians. The control group consisted of 134 school-age children at a school of the same size with their parents**/**guardians.

**Results:**

Results show that nutritional status was significantly improved within the experimental group (*P* value = 0·000) and between groups during follow-up (*P* value = 0·032). Students’ knowledge about obesity and non-communicable chronic diseases (NCD) prevention, as well as physical activity and exercise behaviours, in the experimental group was significantly higher than that in the control group (*P* value = 0·000 and 0·044, respectively). Parents’ perceptions of child obesity and family modelling behaviours in the experimental group were also significantly higher than that in the control group; *P* value = 0·013 and 0·000, respectively).

**Conclusion:**

The community participation program was found to be successful. Not only students, families and schools improved health behaviours and healthy food environments at home and school, but the students’ long-term nutritional status also improved.

School-age obesity is a serious problem leading to a variety of chronic diseases. The percentage of children affected by obesity has more than tripled since the 1970s^(1)^. Data from 2015–2016 show that nearly one in five school-age children aged 6–12 years in developing countries is obese^(2)^. Empirical evidence demonstrates that obesity during the school years strongly leads to a variety of chronic diseases in adulthood with consistently significant risk factors for non-communicable chronic diseases (NCD) such as CVD, metabolic syndrome and type 2 diabetes mellitus^(3–6)^. However, the sustainability of solving the problem of obesity among school-age children to prevent future risk factors for NCD by promoting a healthy environment would be a key factor that should not to be overlooked.

Previous studies^(7–9)^ have clearly indicated that the causes of obesity are multifactorial and complex. The incidence of obesity among school-age children cannot be explained based on the behavioural factors of students alone; rather, the environmental conditions surrounding students could also influence students’ health-related behaviours^(10–12)^. This finding is supported by the ecology of human development by Bronfenbrenner (1979), who developed an ecological system theory (EST) and illustrated that children’s environments affect them in the form of constant reciprocity between individuals and the environment^(13,14)^. In other words, the family environment is a key setting for fostering healthy food intake and exercise for school-age children in the long run^(10,11)^. At the same time, the school acts as a vital platform for improving future health through learning processes and school policy^(6)^. EST can help researchers explain interactions between school-age children and their environments in developing obesity^(8)^ within complex layers of the environment among a set of nested and intertwined structures^(13,14)^.

According to EST by Bronfenbenner (1979), the environment is clarified as five systems, beginning with the environment closest to the individual and extending to a broad distance from the individual. The microsystem describes settings in which there are direct interactions with children. This level is considered to be nearest to the individual and includes such settings as family environments. The home is the starting point for fostering children’s health behaviours because home environments support family members in their efforts to have good health by providing healthy foods, purchasing nutritious and accessible foods for family members, increasing vegetable and fruit consumption and reducing sedentary activity^(15)^.

The mesosystem describes settings in which children are immersed and involves connections between settings, such as links between school and family. The exosystem describes settings in which there are no direct interactions with children, but indirect influences instead, such as school environments. Students spend at least eight hours per day at school^(16)^. Therefore, school could be counted as an important environment to improve knowledge through classroom lessons, promotion of nutritious foods, reduction of unhealthy foods such as sweets and increases of healthy foods such as vegetable and fruit menus during school lunches, together with increases in daily exercise through school policy^(17)^. The macrosystem involves the cultural and political values of a society, economic models and social conditions, and the chronosystem involves the historical moment in which the individual lives. School-age children spend their lives in two main communities, namely home and school^(15)^. Therefore, schools and family members are considered communities for school-age children^(15)^. In-depth understanding of the root causes of school-age obesity might lead to long-term and appropriate solutions in a factual context, which could result in a sustainable impact on the prevalence school-age obesity at the community level^(18)^.

Unfortunately, studies focused on individual-level prevention have been found to be unsuccessful and fail to create sustainability^(16,17)^. Obviously, during school term breaks, the incidence of obesity is higher and continues to rise. Therefore, not only school factors influence school-age obesity, but family environments do, as well. Family factors are concealed with tremendous influence in building sustainability in school-age obesity prevention^(18)^. Previous studies have strongly recommended that family environmental factors must be taken under consideration in fostering health behaviour among students so they can grow up as healthy adults^(19)^. Successful promotion of healthy food environments for long-term obesity and NCD prevention requires solutions covering all three levels at the individual, family and school levels^(5,15,20,21)^. In particular, families need to seriously participate in solving the problem from the start until the end of the process. Participation leads to all parties gaining the same understanding of the problem and setting the same goals^(13,14)^.

Thus, collaboration in problem-solving guidelines is sought in line with the real problems with an enhanced sense of ownership of the problem and solutions^(13,21)^. This collaboration will pave the way for school and family to prevent school-age obesity in the same direction, while contributing to sustainable and policy-driven programmes^(5,22,23)^.

Currently, research on childhood obesity prevention places greater focus on family participation, but only as supplementary efforts under school-based programmes^(24,25)^. Unfortunately, parents in previous studies typically were engaged by passive methods, such as newsletters^(26)^, in one-way communication^(25)^. Not surprisingly, these methods failed to engage parents and improve the nutritional status of children in the long run^(24)^. On the contrary, some studies have tried to promote collaborative efforts between school and family to prevent childhood obesity by emphasising participation principles through identifying problems and finding solutions by involving all stakeholders with bottom-up approaches^(17,21)^. The findings highlighted the development of participatory programmes by school and family collaboration to promote healthy food environments for obesity prevention by means of participatory action research (PAR), which is more likely to reduce limitations regarding sustainability^(5,20,21,27)^.

Development of the programme based on the principle of participatory action is likely to yield positive results^(20,21)^. Unfortunately, few studies have evaluated the effectiveness of programmes that were developed with this method^(20,21)^. Therefore, the researcher’s interest is in developing an obesity prevention programme based on EST by applying PAR with school and families for school-age obesity prevention. Kemmis and Mc Taggart (1990) stated that the PAR process consists of the following four steps: planning (discovering the cause of the problem, knowing the needs of all the stakeholders, solving problems, coping with barriers and applying problem-solving guidelines), action (experimentation in real situations), observation (observing possibilities) and reflection (reviewing and analysing processes and results)^(28)^. We provide an overview of our methodological approach here with more detail in a forthcoming article. This paper highlights an action step regarding the effectiveness of school and family participatory programs to promote healthy food environments for obesity and NCD prevention among school-age children.

## Methods

### Design

A quasi-experimental design was used to test the effectiveness of a community participatory program. Pretest, posttest and follow-up scores were assessed among experiment and control groups.

### Participants

The purposive sampling technique was used in the study in areas among the top five provinces where child obesity is prevalent, one of which was Aong Karuk, Nakhon Nayok Province. Two public primary schools were selected as the experimental and control groups. The two schools are located in Health Region Area 4 with high statistics for children with obesity in Thailand. The schools were also willing to participate. Both schools met the health-promoting school criteria at the gold level under the health-promoting school recommendations of the WHO. There were 138 and 134 school-age children in the experimental and control groups, respectively, who were studying in Grades 2–6 during the first semester of the 2019 academic year, together with their parents/guardians in the same family.

### Inclusion criteria

Students with normal and overweight nutritional status were included.

### Exclusion criteria

Students who transferred schools or dropped out during the study were excluded.

Students with underweight and obese nutritional status were excluded.

### Intervention

**Fig. 1:**
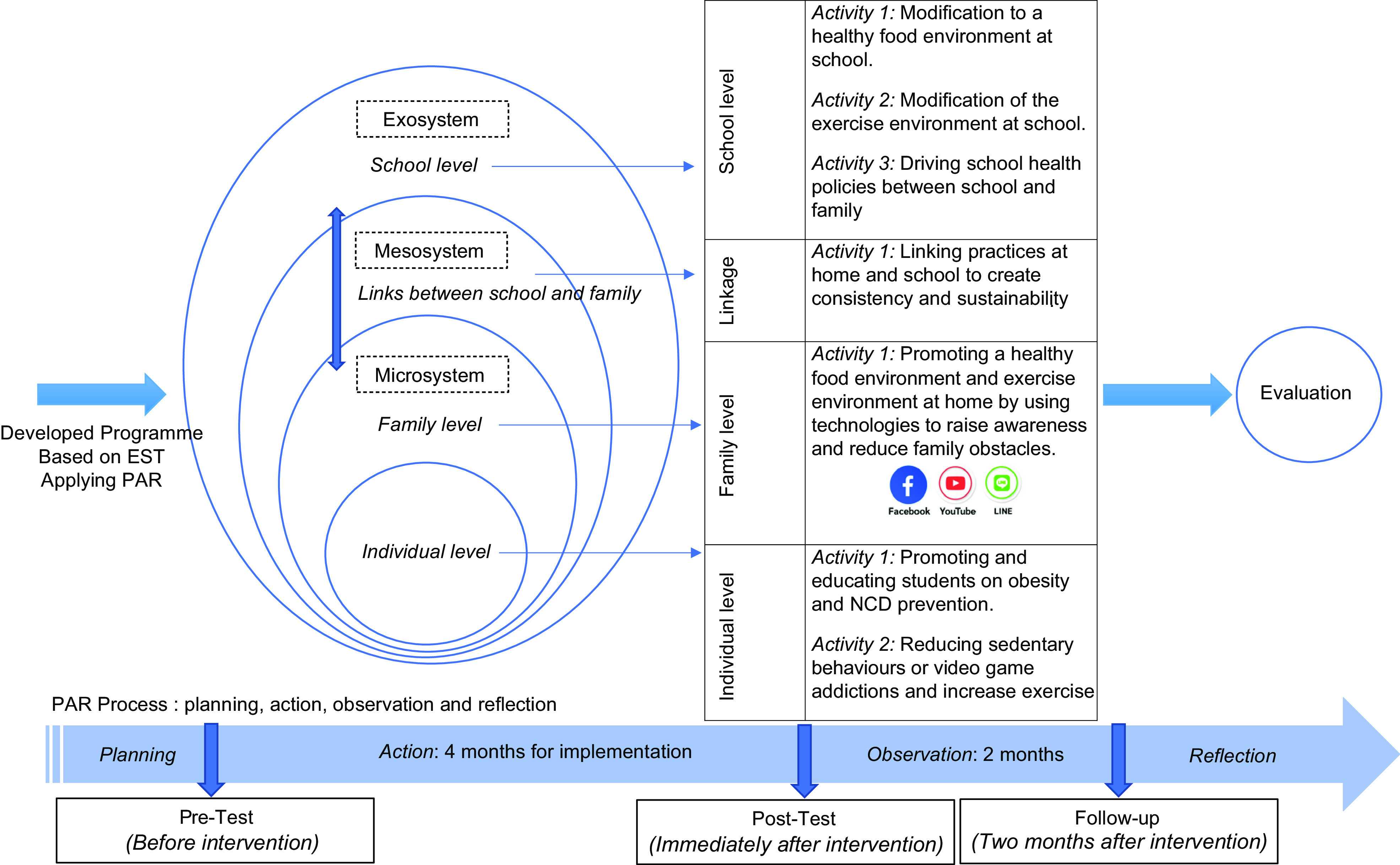
The process of promoting healthy food environments for obesity and non-communicable chronic diseases (NCD) prevention among school-age children

Seven activities, as shown in Fig. 1 and Table 1, were the result of mutual participation in assessment, analysis and decisions made together between family and school. Representatives were appointed from both sides to take responsibility for the planned project. These representatives were called the core working group, which was made of ten willing volunteers composed of six persons from school (school director, two teachers from Grades 4 and 5, two physical education teachers and a school nurse) and 4 persons from a family (of whom one was a public health nurse). Four persons from the core working group later became the research assistants. Student representatives also participated. The role of the researcher was to catalyse and support-related theoretical knowledge.

**Table 1:** Activities and operational plans based on ecological system theory by teachers and parents (June–September 2019; 4 months)

Level	Activities
Individual level activities	Activity 1: Promoting and educating students on obesity and NCD prevention by teachers - Contents on healthy eating and physical activity from teachers who integrated the content with daily lessons - Creating health knowledge boards along with entertaining games for students at school - Monitoring the nutritional status of the students and teaching students to do self-assessments of their nutritional status Activity 2: Reducing sedentary behaviours or video game addictions and increasing exercise - Encouraging students to make beneficial use of free time at school and home
Family level activities (Microsystem)	Activity 1: Promoting of a Healthy Food Environment and Exercise Environment at Home by Use of Technology - Providing knowledge for parents/guardians by using an online course through three online platform channels (YouTube, Facebook, LINE) to raise awareness and reducing family obstacles (co-produce, co-design and co-create informational media via online platform together linking school and family) - Learning from an online course for parents (Healthy-food eating and physical activity, family modelling and parenting practices)
Mesosystem	Activity 1: Linking practices at home and school to create consistency and sustainability - Meeting to discuss health behaviours and healthy food environments of the students at home and school between parents and teachers through the following two activities: 1. Recording daily behaviours at school and home in terms of dietary intake and physical activities; 2. homework on healthy eating and physical activity, family modelling and parenting practices where families put knowledge into practice at home to monitor and control students’ behaviours in line with school activities
School level activities (Exosystem)	Activity 1: Modification to a healthy food environment at school - Changing of school lunches for students from the past when students could make their own purchases to having the school organise a school lunch project for all students (providing highly nutritious foods and vegetable menu items at school for students) - Selling nutritious foods, such as fruits, to provide students with access only to healthy foods at their schools; setting of rules for vendors and holding meetings once per term between teachers and parents**/**guardians to discuss improvements in healthy menu items together Activity 2: Modification of the exercise environment at school - Organising 15-minute aerobic dance activities to a set rhythm in the mornings after lining up for the national anthem - Encouraging students to exercise more and making sports equipment available for students to play with during their free time Activity 3: Driving School Health Policies Together: Create an obesity and NCD prevention policy at school.

NCD, non-communicable chronic diseases.

### Outcomes

#### Measures

To guarantee measurement quality and minimise error, weight and height measurements were double checked before recording. To ensure reliability, the researcher assistants were trained for data collection prior to the study. This study used the Thai Standard Growth Chart weight-for-height ratios for Thai students^(29)^. The Bureau of Nutrition, Ministry of Public Health has developed a standard chart adjusted by weight, height and sex specific to the Thai population from 6 to 19 years of age to define underweight, at risk of underweight, normal weight, at risk of overweight, overweight and obesity conditions^(29)^. However, the current study focused on the primary prevention of obesity for school-age children. Children with obesity (defined as school-age children over +3 sd) and underweight children (defined as school-age children under –2 sd) who had malnutritional status were excluded from the study. Students with normal and overweight nutritional status were invited to join the programme. Normal-weight children ranged from normal weight, at risk of underweight and at risk of overweight, which ranged from –2 sd to +2 sd, while overweight children ranged from +2 sd to +3 sd.

### Output

#### Instrument development and quality testing

The researcher and the core working group developed a set of questionnaires together, and the researcher tested the questionnaires for quality, as follows:

For questionnaires, the content validity index (CVI) is the proportion of items with expert consensus and should not be < 0·80. In the current study, testing was done by presenting the forms to three qualified experts to test for content validity. Next, the questionnaires were pilot tested with thirty school-age children (fifteen normal-weight students and fifteen overweight students) and their families at a school with a similar context. Cronbach’s alpha was calculated to test internal consistency in the pilot study and had to be no < 0·75.

In-depth interview guidelines were followed by the school director, who steered the policy and participant-observation guidelines for the primary researcher to observe monitoring healthy environment factors at school. The advisory professors were asked to examine content accuracy and completeness in addition to making corrections. Next, three qualified experts consisting of a nutrition therapist, a paediatric nutrition expert and a paediatrician expert examined the content validity.

#### Individual-level variables

For individual-level variables, the questionnaires were divided into the following two parts: Part 1 was a questionnaire asking students about their obesity knowledge and perception; and Part 2 was a questionnaire asking students about their physical activities, exercise behaviour and food consumption behaviours. The respondents were Grades 2–6 students aged 8–12 years. The questions were read to the students by the researcher and well-trained research assistants. Nevertheless, behavioural questionnaires were sent to parents**/**guardians for confirmation.

Children’s Perception Questionnaires: six items were asked about children with obesity and prevention, including social norm attitudes on preferable appearance. The six items were scored on a three-point scale with (1) disagree, (2) uncertain and (3) agree. The scale applied cartoon faces to represent the scale range from 1 to 3. This method fit the children’s age group by making the questions interesting and easier to answer. The CVI was 1·0, and Cronbach’s alpha coefficient was 0·79.

Physical Activity and Exercise Behavior Questionnaires: seven items with cartoon pictures of activities asked about physical activities and exercise behaviour during and after school at home. The seven items were scored on a five-point scale with (1) never, (2) seldom, (3) sometimes, (4) often and (5) daily. The CVI was 0·88, and Cronbach’s alpha coefficient was 0·75.

Obesity Related Knowledge Questionnaire: There were ten items that asked about causes of obesity, health effects of obesity, healthy foods and unhealthy foods, sedentary lifestyle and physical activity. The ten items were scored on a true-false scale with (0) false, (0) uncertain and (1) true. The CVI was 0·9, and Kuder–Richardson Formula 20 (KR20) was 0·75.

Food Consumption Questionnaire: twenty items with pictures of various types of snacks to be chosen covered unhealthy and healthy products during and after school at home. The twenty items were scored according to the frequency of dietary intake on a five-point scale with (1) never, (2) seldom, (3) sometimes, (4) often and (5) daily. The CVI was 1·0, and the Cronbach’s alpha coefficient was 0·81.

#### Family-level variables

For family-level variables, the questionnaires were divided into the following three parts: family perception of child obesity, family modelling and parenting practices. The respondents were the same parents**/**guardians or same family members in all families participating in the study.

Family Perception of Child Obesity Questionnaire: There were twelve items that asked about family viewpoints on the nutritional status of their children. The total of twelve items was scored on a four-point scale with (1) totally disagree, (2) disagree, (3) agree and (4) totally agree. The CVI was 0·83, and the Cronbach’s alpha coefficient was 0·80.

Family-Modeling Behavior Questionnaire: There were thirteen items that asked about individual practice as a good role model of healthy eating and exercise^(11)^. The total of twelve items was scored on a five-point scale with (1) never, (2) seldom, (3) sometimes, (4) often and (5) daily. The CVI was 0·85, and Cronbach’s alpha coefficient was 0·80.

Parenting Practices Questionnaire: There were twenty items that asked about the performance of parents in monitoring their children’s food consumption, physical activity and exercise behaviours at home^(30)^. The total of twenty items was scored on a five-point scale with (1) never, (2) seldom, (3) sometimes, (4) often and (5) daily. The CVI was 1·0, and the Cronbach’s alpha coefficient was 0·81.

#### School-level variables

For school-level variables, the data were collected by qualitative data collection techniques with in-depth interview guidelines and participant observation about school environment factors promoting the prevention of obesity.

An in-depth interview was conducted with one school director as a rich, informative case. The primary researcher conducted the interview by using open-ended questions following interviewing guidelines. The interview lasted approximately 1 h and 30 min. All data were audio-recorded, and field notes were taken.

Participant observation was conducted to observe school environment factors comprising the foods provided from the school canteen and foods sold by vendors around the school. The observation was not limited to school heath activities deployed as a result of school health policy and curriculum.

### Statistical analysis

The researcher and the core working group worked together in collecting the quantitative data while the researcher proceeded with the statistically analysis. The quantitative data collection included pretest, posttest (immediately after intervention) and follow-up (2 months after intervention). Quantitative data analysis involved descriptive analysis, independent-sample *t* test, pair *t* test, repeated measures ANOVA and *χ* ^2^ test statistics. Child weight and height measurements were assessed and classified according to the standard sex-specific growth chart on weight and height for Thai citizens for nutritional status^(29)^.

## Results

### Individual level

#### Nutritional status

The comparison points at the three times (pre-test at first week, post-test at the fourth month right after intervention, follow-up at 6 months later in both the experimental and control groups) showed that the nutritional status of the students in the control group did not improve, while the comparison points at the three times in the experimental group showed a nutritional-status progression of the students. As shown in Table 2, the results show that 0 % of students in the experimental group were obese from pretest to posttest with a slight increase to 0·7 % at follow-up, while the prevalence rate of obese students in the control group from pretest to posttest was 0 % to 1·5 %. Furthermore, this rate remained constant (1·5 %) at follow-up. After testing differences between the experimental and control groups, no significant differences were found between the groups at pretest and posttest (*P*-value = 0·728 and 0·144, respectively), while significant differences between the groups were found at follow-up (*P* value = 0·032). Observations were made of the growing negative effects. Even at the beginning, obese students had already been excluded from the study. It seems that during the six-month long intervention, certain factors could have caused some obesity to develop in in the students. Ultimately, the obese students from the control group were higher in number when compared with those of the experimental group.

**Table 2:** Description of students’ nutritional status compared at three time points in the experimental (*n* 138) and control groups (*n* 134)

Nutritional status	Pretest		Posttest		Follow-up	
	*n*	%	*n*	%	*n*	%
Experimental group (*n* 138)						
Underweight	0	0	6	4·3	8	5·8
Normal	120	87·0	123	89·1	122	88·4
Overweight	18	13·0	9	6·5	7	5·1
Obese	0	0	0	0	1	0·7

	Mean	sd	Mean	sd	Mean	sd
Weight	32·00	9·25	33·45	9·47	34·65	9·64
Height	135·88	11·05	137·75	11·03	140·23	11·46
Within group	χ ^Pre × Post^_(df **=** 2)_ = 0·000*		χ ^Pre × Follow up^_(df **=** 3)_ = 0·000*		χ ^Post × Follow up^_(df **=** 6)_ = 0·000*	
Control group (*n* 134)						
Underweight	0	0	4	3·0	6	4·5
Normal	114	85·1	111	82·8	105	78·4
Overweight	20	14·9	17	12·7	21	15·7
Obese	0	0	2	1·5	2	1·5

	Mean	sd	Mean	sd	Mean	sd
Weight	35·40	11·43	36·34	12·28	37·76	12·42
Height	136·82	10·95	139·37	11·56	140·81	11·59
Within group	χ ^Pre × Post^_(df **=** 2)_ = 0·000*		χ ^Pre × Follow up^_(df **=** 3)_ = 0·000*		χ ^Post × Follow up^_(df **=** 9)_ = 0·000*	
Between groups	χ† ^Pretest^ = 0·728		χ ^Posttest^_(df = 3)_ = 0·144		χ ^Follow up^_(df = 3)_ = 0·032*	

df, degree of freedom.

Pre × Post **=** compare differences between pretest and posttest.

Pre × Follow-up **=** compare differences between pretest and follow-up.

Post × Follow-up **=** compare differences between posttest and follow-up.

* *P* value < 0·05.

† Fisher’s exact test.

#### Physical activity and exercise behaviours

The scores for physical activity and exercise behaviours in the experimental group were significantly higher than that for the control group (F_(1,270)_ = 28·388; *P* value = 0·000). The results showed no differences at pretest, but significant differences between posttest and follow-up (*P*-value = 0·456, 0·000 and 0·018, respectively). However, the results found no significant differences within the experimental group (F_(1,1·907)_ = 2·693; *P*-value = 0·071). (See Tables 3 and 4)

**Table 3:** Comparison of physical activity and exercise behaviours of students between groups and within groups

Source of variables	SS	df	MS	F	*P* value*,†
Between groups					
Groups	10·475	1	10·475	28·388	0·000*
Between-group error	99·630	270	0·369		
Within groups					
Time	1·926	1·907	1·010	2·693	0·071
Time × Groups	5·372	1·907	2·817	7·512	0·001*
Within-group error	193·063	514·835	0·375		

SS, sum of square; df , degree of freedom; MS, mean of square.

* *P* value < 0·05.

† Greenhouse-Geisser.

**Table 4:** Mean scores for the physical activity and exercise behaviours of students at three time points in the experimental and control groups†

Physical activity and exercise behaviours	Experimental group (138)		Control group (134)				
	Mean	sd	Mean	sd	t	df	*P* value*
Pretest	3·10	0·61	3·05	0·59	0·747	270	0·456
Posttest	3·23	0·61	2·79	0·53	6·419	270	0·000*
Follow-up	3·22	0·64	3·04	0·62	2·372	270	0·018*

* *P* value < 0·05.

† A mean score of 1–3 points means ‘behaviour needs improvement’, a mean score of 3·1–3·95 points means ‘moderate level of behaviour’ and a mean score of 4·0–5·0 points means ‘good level of behaviour’.

Students’ Knowledge about Obesity and NCD prevention – The scores for students’ knowledge about obesity and NCD prevention in the experimental group were significantly higher than in the control group (F_(1,270)_ = 4·090; *P* value = 0·044). The results showed no differences between pretest and posttest, but significant differences at follow-up (*P* value = 0·947, 0·362 and 0·005, respectively). In addition, the results found significant differences within the experimental group (F_(1,1·829)_ = 7·806; *P* value = 0·001). Scores were significantly higher from pretest to posttest, posttest to follow-up and pretest to follow-up (*P* value =< 0·007, 0·001, 0·001, respectively) (see Tables 5–7).

**Table 5:** Comparison of students’ knowledge about obesity and non-communicable chronic diseases (NCD) prevention between groups and within groups

Source of variables	SS	df	MS	F	*P* value*,†
Between groups					
Groups	19·094	1	19·094	4·090	0·044*
Between-group error	1260·360	270	4·668		
Within groups					
Time	43·971	1·829	24·041	7·806	0·001*
Time × Groups	20·057	1·829	10·966	3·560	0·033*
Within-group error	1520·784	493·761	3·080		

SS, sum of square; df, degree of freedom; MS, mean of square.

* *P* value < 0·05.

† Greenhouse-Geisser.

**Table 6:** Mean scores for knowledge about obesity and non-communicable chronic diseases (NCD) prevention at three time points between the experimental and control groups†

Knowledge of students	Experimental group (138)		Control group (134)				
	Mean	sd	Mean	sd	t	df	*P* value*
Pretest	7·69	1·65	7·70	1·57	−0·067	270	0·947
Posttest	8·34	1·94	8·14	1·63	0·913	270	0·362
Follow-up	8·47	1·92	7·74	2·30	2·849	270	0·005*

* *P* value < 0·05.

† A mean score of 8–10 points was interpreted as ‘high’ (> 80 %), a mean score of 6–7 points was interpreted as ‘moderate’ (60–79 %) and a mean score of 0–5 points was interpreted as ‘low’ (< 60 %).

**Table 7:** Comparison of students’ knowledge about obesity and non-communicable chronic diseases (NCD) prevention at three time points between the experimental and control groups

Students’ knowledge	Mean difference	Std. error	*P* value*,†
Experimental group			
Posttest – Pretest	0·652	0·211	<0·007*
Follow-up – Posttest	0·130	0·034	<0·001*
Follow-up – Pretest	0·783	0·208	<0·001*
Control group			
** **Posttest – Pretest	0·440	0·189	<0·064
Follow-up – Posttest	−0·403	0·251	<0·331
Follow-up – Pretest	0·037	0·252	<1·000

* *P* value < 0·05.

† Bonferroni’s method.

### Family level

#### Parents’ perception

The scores for parents’ perceptions about obesity and NCD prevention in the experimental group were significantly higher than for the control group (F_(1,270)_ = 6·191; *P* value = 0·013). The results showed that there were no differences at pretest and posttest, but significant differences at follow-up (*P* value = 0·760, 0·157 and 0·000, respectively). In addition, the results found significant differences within the experimental group (F_(1,1·662)_ = 19·460; *P* value = 0·000). Scores were significantly higher from pretest to posttest, from posttest to follow-up and from pretest to follow-up (*P* value =< 0·000, 0·065, 0·000, respectively) (see Tables 8–10).

**Table 8:** Comparison of the parents’ perceptions about obesity in their children between groups and within groups

Source of variables	SS	df	MS	F	*P* value*,†
Between groups					
Groups	153·517	1	153·517	6·191	0·013*
Between-group error	6694·650	270	24·795		
Within groups					
Time	510·758	1·662	307·315	19·460	0·000*
Time × Groups	180·425	1·662	108·559	6·874	0·002*
Within-group error	7084·433	448·609	15·792		

SS, sum of square; df, degree of freedom; MS, mean of square.

* *P* value < 0·05.

† Greenhouse–Geisser.

**Table 9:** The average mean scores of the parents’ perceptions about obesity in their children at three time points in the experimental and control groups†

Parents’ perception score	Experimental group (138)		Control group (134)				
	Mean	sd	Mean	sd	t	df	*P* value*
Pretest	34·23	4·07	34·39	4·35	−0·306	270	0·760
Posttest	36·17	3·73	35·52	3·75	1·420	270	0·157
Follow-up	37·16	4·54	35·04	4·24	3·967	270	0·000*

* *P* value < 0·05.

† A mean score of 12–23 points was interpreted as ‘poor’, a mean score of 24–35 points was interpreted as ‘moderate’ and a mean score of 36–45 points was interpreted as ‘good’.

**Table 10:** Comparison of the parents’ perceptions about obesity in their children at three time points between the experimental and control groups

Parents’ perceptions	Mean difference	Std. error	*P* value*,†
Experimental group			
Posttest – Pretest	1·935	0·221	<0·000*
Follow-up – Posttest	0·993	0·427	<0·065
Follow-up – Pretest	2·928	0·506	<0·000*
Control group			
Posttest – Pretest	1·134	0·423	<0·025*
Follow-up – Posttest	−0·478	0·480	0·964
Follow-up – Pretest	0·657	0·517	0·618

* *P* value < 0·05.

† Bonferroni’s method.

#### Family modelling

The scores for family modelling in the experimental group were significantly higher than the control group (F_(1,270)_ = 13·858; *P* value = 0·000). The results showed no difference at pretest but significant differences between posttest and follow-up (*P* value = 0·108, 0·000 and 0·003, respectively). In addition, the results found significant differences within the experimental group (F_(1,1·941)_ = 12·610; *P* value = 0·000). However, there were no significant differences between pretest and posttest, but scores were significantly higher from posttest to follow-up and from pretest to follow up (*P* value =< 0·474, 0·050, 0·002, respectively) (see Tables 11–13).

**Table 11:** Comparison of family modelling between groups and within groups

Source of variables	SS	df	MS	F	*P* value*,†
Between groups					
Groups	4·961	1	4·961	13·858	0·000*
Between-group error	96·660	270	0·358		
Within groups					
Time	7·539	1·941	3·884	12·610	0·000*
Time × Groups	8·880	1·941	4·575	14·854	0·000*
Within-Group error	161·396	524·014	0·308		

SS, sum of square; df, degree of freedom; MS, mean of square.

* *P* value < 0·05.

† Greenhouse–Geisser.

**Table 12:** Mean scores of family modelling at three time points in the experimental and control groups†

Family godeling	Experimental group (138)		Control group (134)				
	Mean	sd	Mean	sd	t	df	*P* value*
Pretest	3·50	0·66	3·63	0·60	−1·610	270	0·108
Posttest	3·61	0·54	3·23	0·36	6·822	239·173	0·000*
Follow-up	3·76	0·60	3·54	0·57	2·973	270	0·003*

* *P* value < 0·05.

† A mean score of 1–3 points means ‘behaviour needs improvement’, a mean score of 3·1–3·95 points means ‘moderate level of behaviour’ and a mean score of 4·0–5·0 points means ‘good level of behaviour’.

**Table 13:** Comparison of family modelling at three time points between the experimental and control groups

Parents’ perceptions	Mean difference	Std. error	*P* value*,†
Experimental group			
Posttest – Pretest	0·102	0·072	<0·474
Follow-up – Posttest	0·149	0·062	<0·050
Follow-up – Pretest	0·251	0·072	<0·002*
Control group			
Posttest – Pretest	−0·400	0·059	<0·000*
Follow-up – Posttest	0·316	0·061	<0·000*
Follow-up – Pretest	−0·084	0·070	<0·691

* *P* value < 0·05.

† Bonferroni’s method.

### School level

From in-depth interviews and participant observation, it was found that school health policy became more active. Health promotion and policies for preventing obesity among children were integrated with school lessons in subjects such as physical education, health education and science, all of which involve long-term activities. Furthermore, there was an initiative in team setting for school projects on preventing obesity in children. The initiative team typically came from the research core working group. Moreover, the Association of Leading Parents was appointed to work in school health networking. The parent team was very strong and helpful in taking policy into in the long term. Unhealthy food vendors were banned both from school itself and from the areas surrounding the school. The school launched the school lunch project suggested by the Ministry of Public Health so that students would receive highly nutritious foods, avoid junk food and add vegetable and fruit items to their menus. Regular exercise was scheduled 15 min every morning before class. The school improved facilities and sports areas for recreation to increase space for physical activities and exercise. The facilities for physical activities and exercise were also improved.

## Discussion

The programme was found to be successful in terms of improving health behaviours and environments both at home and at school, as suggested by EST. Students’ nutritional status showed the progression of students with normal weight with likelihood of future sustainability as a result of true collaboration in the PAR process.

In terms of EST at the individual level, the results showed improvement in physical activity, exercise behaviours and knowledge about obesity and NCD prevention among students. This finding corresponds with the findings of previous studies that were focused on promoting exercise in school-age children^(31)^ and programmes that were focused on knowledge about food and nutrition and which were combined with promoting afterschool exercise^(32)^. The findings indicate that promoting exercise at school is efficient in improving the overall physical fitness of students^(31)^. In addition, it helps with improvements in four healthy eating behaviours: fruits, vegetables, sugar-free beverages and avoidance of unhealthy snack foods^(32)^.

For the microsystem, the results revealed that the families had changed in terms of their perceptions of obesity in children and family modelling behaviours in their efforts to promote healthy children. Although the results show no significant difference in parenting practices between the experimental and control groups, the qualitative data show that parents are prone to be more concerned about healthy food selection, sedentary reduction and regular exercise. This study was supported by empirical evidence indicating that parents as role models are associated with young children’s weight, dietary intake and physical activity^(33)^.

For the exosystem, the qualitative results revealed that there were noticeable improvements in school environments in the aspects of health-promoting policy and curriculum integrated at school. In particular, the improvement in the healthy food environments included physical activity and exercise facilities at school. Three significant changes were made in the school environment: (1) integration of obesity and NCD prevention knowledge in the course curriculum and (2) creation of healthy policies related to obesity prevention such as modification of school lunch projects and healthy food vendor management. The school’s chef was trained in nutritionally based healthy cooking under the recommendations of the Ministry of Public Health. This policy of healthy cooking is consistent with the findings of Li *et al.* ^(34)^, who affirmed that issuing rules for food sales in school cafeterias was correlated with reducing the intake of energy-dense foods and the prevalence of overweight conditions and obesity among school-age children. Ensuring that the school environment is filled with more healthy than unhealthy foods led to modifications in student consumer behaviours with access to healthy foods.^(35)^ (3) Adding an hour for exercise and facilitating access to sports equipment and playground areas in this study correlated well with a study done by Cheng *et al.* ^(36)^, who found school physical activity environments to be associated with students’ obesity with statistical significance, including duration of physical activity during school for at least 1 h**/**d and the provision of playground equipment such as climbing structures and swings. Adequate daily exercise, access to sports and playground equipment may, therefore, facilitate school-age obesity prevention^(36,37)^.

Accordingly, times are always changing. The culture of a society and social conditions have changed from the traditional Thai food culture. Western-style fast-food consumption (saturated fat and sweet drink intake) plays an important role in Thai daily life. School-age children now follow fashion**/**trends in food consumption because it represents modernity and such foods are easy to buy.

Previous research has illustrated interesting issues concerning health programmes aimed at preventing obesity in school-age children and how real participation between family and school to make changes in both home and school environments together can lead to individual behaviour modification^(10,21)^. These factors are the key to success, as they lead to satisfaction on all sides and result in cooperative problem solving that is sustainable^(5,38)^. Previous research has also found that focusing on only one side (home or school) or only on individual behaviour modification results in less likelihood of long-term behaviour modification, as observed in the absence of improvement in the students’ nutritional status^(32,39)^. The reason for this finding may result from the fact that the activities focused only on school practice and did not include continuing practice at home^(22,38,39)^.

The outstanding features of the current study are as follows: (1) the study was based on EST with intervention efforts covering individual, family and school levels; The problem of obesity in school-age children is complex, and previous findings have yielded vague problem-solving guidelines, whereas sustainable solutions are required for preventing obesity in school-age children. It is anticipated, therefore, that this task would remain difficult if it continued towards separate solutions between school and family or opted to solve the problem of obesity in school-age children only at school or at home because one factor may influence others^(40)^. Weight control within a normal range for children must rely on promotion by family and school. This need for family and school involvement means that interaction at the family and school levels can have tremendous influence over long-term changes in behaviour^(9,21)^. This finding corresponds with the findings of previous studies in which it has been stated that preventing childhood obesity cannot be aimed at only one aspect. Adjusting both home and school environments together can result in consistency and continuity of practice, both at home and at school. Making this adjustment can lead to sustainability in preventing obesity in school-age children^(12)^ and strongly suggests that disconnected practice between school and home can only adversely affect the sustainability of solutions^(41)^.

(2) The programme was developed by applying the PAR process from beginning to end. The results yielded by application of the PAR process in this study both support and confirm the statements of previous researches that applied the PAR process to create numerous benefits for the participants: (1) every party had the same goal of reducing the problem of misunderstanding^(20,21)^; (2) empowerment was created among all stakeholders, which served as a key foundation for future extension^(5)^ and (3) participation was increased at every stage and every party was instructed to feel love, attachment and ownership of the problem together^(13,14)^. The PAR process adhered to the principle of participation by all parties and promoted motivation for every party to participate in offering opinions or proposing ideas while sharing experiences and knowledge together^(23–41)^. This study also found that adhering to the principles of a participatory process based on EST from beginning to end of the process resulted in work-performance clarity, reduced the problem of confusion and built uniform understanding among all parties. Nevertheless, the main barrier found during the course of conducting the study was that families had no time to participate in all school activities because of time constraints caused by working to earn a living^(41–44)^. Therefore, families proposed the use of technology to help reduce low participation and to improve the communication linkage between school and family. This study is supported by previous research stating that applying these technologies enhances strong participation and linkage between school and family^(45)^. Moreover, the collaboration between school and family to design child obesity prevention video clips resulted in media that met the needs and was more interesting to follow^(46)^.

The purpose of this study was to formulate policies for controlling obesity among school-age children. These policies were found to be more effective because (1) good relationships were built, which led to a mutual willingness to hear opinions and needs of each party^(20–22,42)^; (2) opportunities were offered for every party to participate in the problem-solving process, which led to new ideas^(22,44)^; (3) parents**/**guardians and teachers were empowered; such empowerment gave every party confidence in solving their problems^(21)^ and (4) family and school felt a joint responsibility, which can result in the creation of sustainable policies in the future^(21)^. In terms of context, this type of intervention is feasible in low^(47)^, middle-income^(20,21)^ and high-income countries^(22)^.

Nevertheless, there were some limitations in this study. First, barriers might be encountered concerning access to technologies by elders and time constraints for family participation because of the need of parents**/**guardians to work and support their families^(20,21)^. Therefore, planning strategies for family participation must be given primary consideration and not be overlooked. Second, during the PAR process, there were some difficulties among stakeholders in terms of making the exact dates for appointments. This situation might have caused them to some extend the delays in their schedules^(43)^. Third, the platforms for disseminating information suggested by the stakeholders included Facebook groups, LINE groups and a YouTube Channel, all of which were considered highly beneficial communication channels. However, the limitation in terms of the process of validation with the target audience could lead to a limitation of the study regarding its methodological aspects. In particular, families with elders would be less likely to be familiar with such platforms. This limitation involved the potential of exclusion and abandonment from their inability to use such platforms. However, a reflection of results resulting during the evaluation period verified the conclusion that it would be of greater benefit if only one platform or technologies was used rather than many. Fourth, it would be better to remove some of the take-home message activities to make the dissemination more practical. Although the results of this study found positive impact in preventing obesity among school-age children, the PAR process in this study included only one loop because of the time and resource constraints of the research.

## Conclusions

This study reveals strategic methodologies for obesity prevention among school-age children to meet the need of encompassing environmental linkages from individual, family and school levels. The investigation that was conducted revealed the programme to be successful and led to improvements in health behaviours and environments. The students’ nutritional-status outcomes indicated successful progression with a likelihood of future sustainability. Developing community participation programmes based on the EST and PAR process provides suitable guidance for future obesity prevention. Nevertheless, students served as excellent bridges as a linkage between home and school. The outstanding strategies involved applying social media platforms as communication channels leading to increased participation.

## Supplementary material

For supplementary material accompanying this paper visit https://doi.org/10.1017/S136898002300040X. click here to view supplementary material
