# Supplementary material for: Ecological system theory and community participation to promote healthy food environments for obesity and non-communicable diseases prevention among school-age children
Source: Public Health Nutr. 2023 Feb 27;26(7):1488–500. doi: 10.1017/S136898002300040X (PMC10346019; doi:10.1017/S136898002300040X)
Supplement: Supplementary file 1 [file S136898002300040Xsup001.docx]

**Questionnaire for Students**

1.Children’s Perception about Obesity in Children and Prevention Questionnaire.

| **Children’s Perception** | **Level of Knowledge**  **(No. and Percentage)** | | |
| --- | --- | --- | --- |
|  | **Agree** | **Uncertain** | **Disagree** |
| 2. I feel like chubby kids are healthy, strong and cute. | ☺ | 😐 | ☹ |
| 3. I feel like most students with obesity from a young age get thinner as they grow up. | ☺ | 😐 | ☹ |
| 1. I feel like school-age kids eat a lot…the amounts are unlimited, especially high-calorie foods like fried foods and sweets, to promote rapid growth. | ☺ | 😐 | ☹ |
| .  .  6. ………………………. |  |  |  |

2.Student Knowledge about Obesity and NCDs Prevention Questionnaire.

| **Question** | **True √** | **False X** | **Uncertain** |  |
| --- | --- | --- | --- | --- |
|  |  |  |  |  |
| 1.Obesity in students is caused by eating too much food. |  |  |  |  |
| 2.Obesity causes heart disease, diabetes and high blood pressure. |  |  |  |  |
| 3.Frequently eating fried foods is **NOT** a risk for obesity. |  |  |  |  |
| 4.Regular exercise can prevent obesity. |  |  |  |  |
| 5. Sitting and playing computer games for 5-6 hours every day **REDUCES** the risk for obesity. |  |  |  |  |
| 6………  .  .  10…… |  |  |  |  |

3.Food Consumption Behavior Questionnaire

| **Food Type** | **Frequency of Food Consumption** | | | | |
| --- | --- | --- | --- | --- | --- |
|  | **Daily** | **Often**  **(4-6 times per week)** | **Sometimes**  **(1-3 times per week)** | **Seldom**  **( 2-3 times per month)** | **Never** |
| 1. I drink flavored, sweetened milk like:  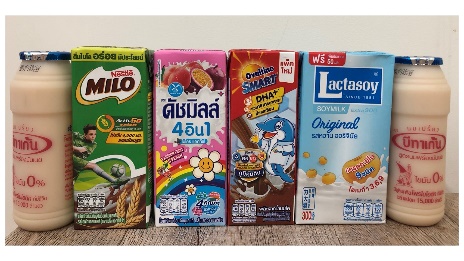 |  |  |  |  |  |
| 2. I eat fast foods and fried foods like:  .........................................  3. I eat crispy snacks like: .........................................  .  .  20................................... |  |  |  |  |  |

4. Physical Activity and Exercise Behavior Questionnaire.

| **Physical Activity and Exercise Behaviors** | **Frequency of Physical Activity or Exercise** | | | | |
| --- | --- | --- | --- | --- | --- |
|  | **Daily** | **Often**  **(4-6 times per week)** | **Sometimes**  **(1-3 times per week)** | **Seldom**  **( 2-3 times per month)** | **Never** |
| 1. During lunch breaks at school, students play on their telephones,  I-Pads or other computers.  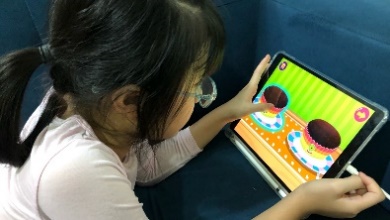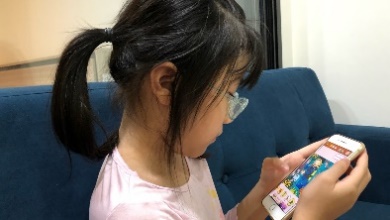 |  |  |  |  |  |
| 2. During school lunch breaks, students run around or play sports with friends.  3. In the evenings after school, students run around or play sports with friends.  .  .  7………………………… |  |  |  |  |  |

**Questionnairs for Parents/Guardians**

1.Family Perceptions about Child Obesity Questionnaires

| **Perceptions about Nutritional Status in School-Age Children** | **Level of Perception (No. and Percentage)** | | | |
| --- | --- | --- | --- | --- |
|  | **Totally Agree** | **Agree** | **Disagree** | **Totally Disagree** |
| 1. Do you think your child/grandchild with obesity is at risk for obesity as he/she grows into adulthood. |  |  |  |  |
| 2. Do you think you should arrange high-calorie foods as wanted by your child/grandchild when he/she gets hungry between meals? |  |  |  |  |
| 2. Do you think the foods you’ve recently prepared for your child/grandchild have affected his/her current health? |  |  |  |  |
| 3. Do you think your child/grandchild with obesity will automatically have a proportionate figure by not having to control food intake and/or exercise when he/she grows up? |  |  |  |  |
| 4.  .  .  .  12................................ |  |  |  |  |

2. Family Modeling Behavior Questionnaire

| **Family Modeling Behavior** | **Frequency of Activities with Child/Grandchild** | | | | |
| --- | --- | --- | --- | --- | --- |
|  | **Daily** | **Often**  **(4-6 times per week)** | **Sometimes**  **(1-3 times per week)** | **Seldom**  **( 2-3 times per month)** | **Never** |
| **Good Family Modeling Behavior in Food Consumption**  1. You only eat enough to feel full and advise your child/grandchild to do the same. |  |  |  |  |  |
| 2. You seldom eat sweet fruits or only eat them in small amounts and select fruits that are not highly sweet such as guavas, dragon fruits, apples and rose apples, etc., and advise your child/grandchild to do the same.  .  8……………………. |  |  |  |  |  |
| **Good Modeling Behavior in Exercise** |  |  |  |  |  |
| 9. You watch TV/play video games/search the Internet on your PC/smart phone/i-Pad as a means of relaxation for long periods of time and allow your child/grandchild to do the same with no time limitations.  .  13. |  |  |  |  |  |

3. Parenting Practice Questionnaire

| **Parenting Practice** | **Frequency of Activities with Child/Grandchild** | | | | |
| --- | --- | --- | --- | --- | --- |
|  | **Daily** | **Often**  **(4-6 times per week)** | **Sometimes**  **(1-3 times per week)** | **Seldom**  **( 2-3 times per month)** | **Never** |
| **Food Consumption**   1. Do you prepare vegetables and fruits to make it **convenient** for your child/grandchild to eat at each meal? For example, do you wash, peel and cut them into small pieces, etc.? |  |  |  |  |  |
| 1. Do you prepare vegetables and fruits to make it **easy** for your child/grandchild **to access** and each whenever he/she wants to? For example, do you cut them into small pieces and place them on a lower shelf of the refrigerator for them to easily grab and eat or do you leave them on the table where they are readily visible, etc.?   .  16…………………………………. |  |  |  |  |  |
| **Physical Activity and Exercise**  17. Do you always support sports equipment such as the preparation of sports equipment and clothing for your child/grandchild, etc.?  18.  .  20. |  |  |  |  |  |
